# Arginase 2 promotes neurovascular degeneration during ischemia/reperfusion injury

**DOI:** 10.1038/cddis.2016.295

**Published:** 2016-11-24

**Authors:** Esraa Shosha, Zhimin Xu, Harumasa Yokota, Alan Saul, Modesto Rojas, R William Caldwell, Ruth B Caldwell, S Priya Narayanan

**Affiliations:** 1 Vascular Biology Center, Medical College of Georgia, Augusta University, Augusta, GA, USA; 2 Vision Discovery Institute, Augusta University, Augusta, GA, USA; 3 Charlie Norwood VA Medical Center, Augusta, GA, USA; 4 Department of Ophthalmology, Asahikawa Medical University, Asahikawa, Japan; 5 Department of Ophthalmology, Augusta University, Augusta, GA, USA; 6 Department of Pharmacology and Toxicology, Augusta University, Augusta, GA, USA; 7 Department of Occupational Therapy, College of Allied Health Sciences, Augusta University, Augusta, GA, USA

## Abstract

Retinal ischemia is a major cause of visual impairment and blindness and is involved in various disorders including diabetic retinopathy, glaucoma, optic neuropathies and retinopathy of prematurity. Neurovascular degeneration is a common feature of these pathologies. Our lab has previously reported that the ureahydrolase arginase 2 (A2) is involved in ischemic retinopathies. Here, we are introducing A2 as a therapeutic target to prevent neurovascular injury after retinal ischemia/reperfusion (I/R) insult. Studies were performed with mice lacking both copies of A2 (A2^−/−^) and wild-type (WT) controls (C57BL6J). I/R insult was conducted on the right eye and the left eye was used as control. Retinas were collected for analysis at different times (3 h–4 week after injury). Neuronal and microvascular degeneration were evaluated using NeuN staining and vascular digests, respectively. Glial activation was evaluated by glial fibrillary acidic protein expression. Necrotic cell death was studied by propidium iodide labeling and western blot for RIP-3. Arginase expression was determined by western blot and quantitative RT-PCR. Retinal function was determined by electroretinography (ERG). A2 mRNA and protein levels were increased in WT I/R. A2 deletion significantly reduced ganglion cell loss and microvascular degeneration and preserved retinal morphology after I/R. Glial activation, reactive oxygen species formation and cell death by necroptosis were significantly reduced by A2 deletion. ERG showed improved positive scotopic threshold response with A2 deletion. This study shows for the first time that neurovascular injury after retinal I/R is mediated through increased expression of A2. Deletion of A2 was found to be beneficial in reducing neurovascular degeneration after I/R.

Retinal ischemia is a major cause of visual impairment and blindness and is involved in various disorders including diabetic retinopathy, acute glaucoma and retinopathy of prematurity. These pathologies share common features including oxidative stress, neurodegeneration, inflammation, activation of glial cells and vascular damage.^1, 2, 3, 4, 5, 6^ Retinal ischemia/reperfusion (I/R) injury models have been widely used to study the mechanisms of neuronal and vascular damage in ischemic retinopathy.^4, 7, 8^ I/R injury occurs upon restoration of tissue blood supply after a period of ischemia. Although there has been much emphasis on studying protective measures to reverse or reduce I/R insult-mediated tissue damage, so far, there is no clinically effective treatment. This is mainly because the molecular mechanisms by which neurovascular dysfunction and injury happen are far from clear.

Degeneration of neurons and increased vascular permeability have been reported after I/R injury.^7, 9, 10^ Our group has shown that activity of the superoxide-generating enzyme NOX2 NADPH oxidase is crucially involved in I/R-mediated neuronal damage in retina.^11^ Studies from our laboratory also have shown a significant role for the urea cycle enzyme, arginase, in mediating neuronal and vascular injuries in other retinal disease models.^12, 13, 14, 15^

Arginase exists in two isoforms, arginase 1 (A1) and arginase 2 (A2).^16^ A1, the cytosolic isoform, is strongly expressed in the liver^17^ and A2, the mitochondrial isoform, is expressed primarily in extra-hepatic tissues, especially the kidney.^18^ Both isoforms are also found in other tissues including brain and retina^12, 19, 20^ and have been linked to diseases, such as hypertension, aging, I/R injury in heart and kidney, and diabetes complications.^21, 22, 23^ Arginase has been extensively studied as a key factor in I/R injury in different organs including liver, heart and kidney.^24, 25, 26^ Studies in models of myocardial and hepatic ischemia/reperfusion injury have shown a beneficial role of arginase inhibition by different agents.^25, 27, 28, 29, 30^ Although less is known about the role of arginase in I/R injury in brain, studies in a model of Alzheimer's disease (AD) have shown that treatment with an inhibitor of arginase protected mice from AD-like pathology.^31^ Upregulation of A2 has also been implicated in vascular dysfunction associated with aging, obesity and retinopathy.^19, 20, 32, 33^ Previous reports have shown that A2 expression increases after traumatic brain injury (TBI) and that TBI-induced impairment of cerebral blood flow is prevented by A2 deletion.^34^ These studies along with our previous studies underscore a crucial role for A2 in central nervous system injury. In this study, we investigated the role of A2 in retinal neurovascular injury following I/R insult.

## Results

### Increased A2 expression during retinal I/R injury

**Figure 1:**
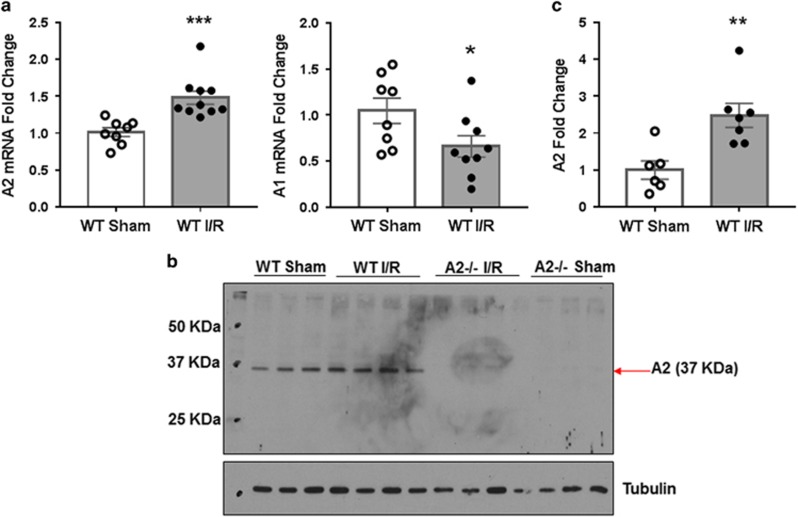
Increased expression of A2 following I/R injury. (**a**) qRT-PCR showing increased A2 mRNA levels and decreased A1 mRNA levels 3 h after I/R in WT retinas. Results are presented as a fold change of WT sham. ***P<0.001 *versus* WT sham, *P<0.05 *versus* WT Sham. For A2 mRNA studies, WT sham: *n*=8 and WT I/R: *n*=10. For A1 mRNA studies, WT sham: *n*=8 and WT I/R: *n*=9. Data are presented as mean±S.E.M. (**b**) Western blot analysis with quantification (**c**) showing increased A2 protein levels in WT retinas 3 h after I/R injury. The red arrow points to the band of interest. Other bands are nonspecific. Molecular size marker is shown on the left. Results are presented as a fold change of WT sham after normalizing to loading control (tubulin). No A2 protein was detected in A2^−/−^ retinas. **P<0.01 *versus* WT sham. WT sham: *n*=6 and WT I/R: *n*=7. Data are presented as mean±S.E.M.

Our previous studies in a model of oxygen-induced retinopathy (OIR) have shown that retinal neurovascular injury is associated with increases in A2 expression and that deletion of A2 limits both neuronal and vascular injuries.^13, 14, 15^ Based on these findings, we hypothesized that A2 is involved in I/R-induced retinal injury. In order to examine this hypothesis, we used quantitative RT-PCR and western blot analyses to examine arginase expression after I/R or sham injury. These studies showed A2 mRNA and protein levels were significantly increased within 3 h after I/R as compared with the sham control (Figures 1a and c). In contrast, levels of A1 mRNA (Figure 1a) and protein (data not shown) were slightly reduced at this time.

**Figure 2:**
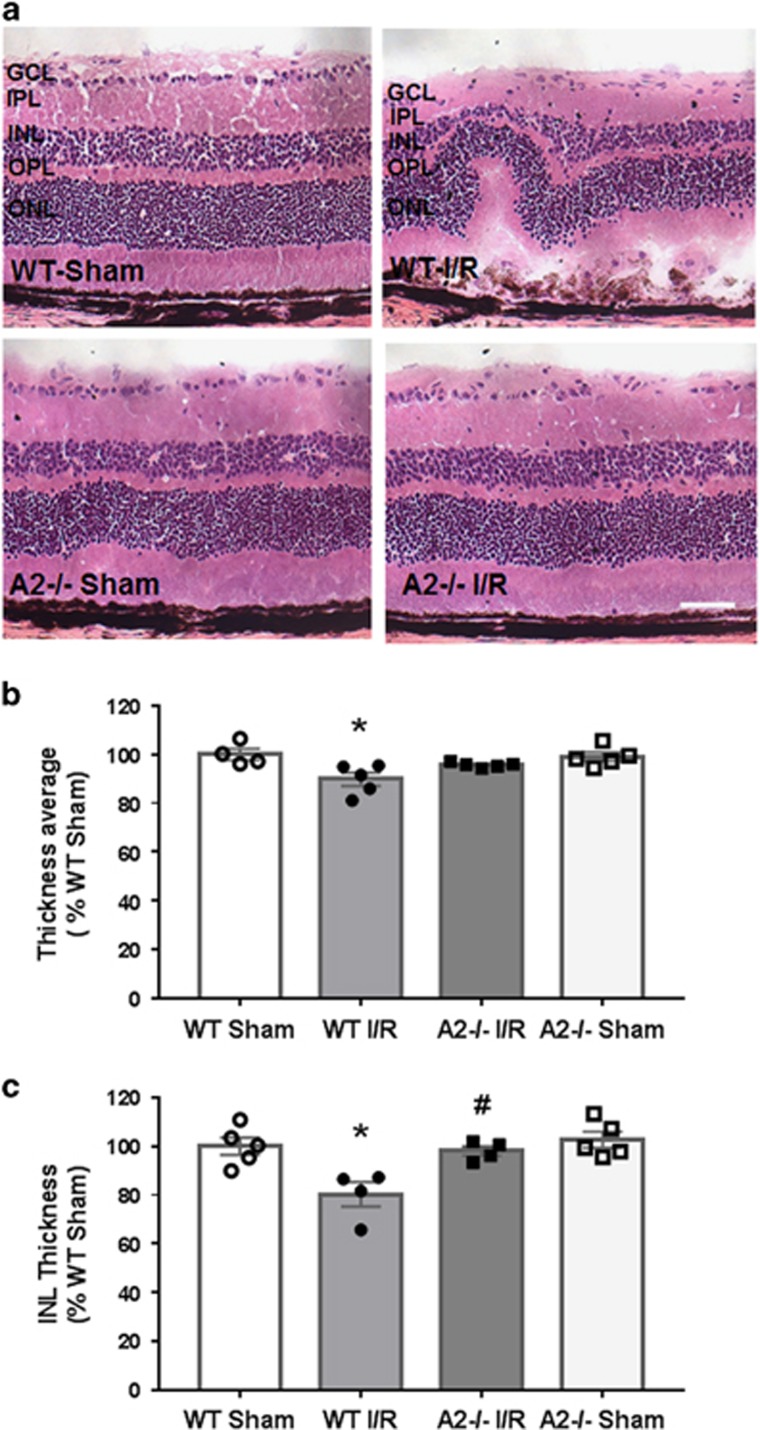
A2 deletion prevented I/R injury-induced retinal thinning. (**a**) H&E images of frozen retinal sections shows loss of cells in the GCL and INL at 7 days after I/R injury. WT sham: *n*=4, *n*=5 per group for others. Scale bar 50 *μ*M. IPL, inner plexiform layer; OPL, outer plexiform layer; ONL, outer nuclear layer. (**b**) Quantification of total retinal thickness. Results are presented as a percentage of WT sham. *P<0.05 *versus* WT sham. WT sham: *n*=4, *n*=5 per group for others. Data are presented as mean±S.E.M. (**c**) Quantification of INL thickness. Results are presented as a percentage of WT sham. *P<0.05 *versus* WT sham, ^#^*P*<0.05 *versus* WT I/R. WT sham and A2^−/−^ Sham: *n*=5, WT I/R and A2^−/−^ I/R: *n*=4. Data are presented as mean±S.E.M.

Along with others, we have shown that I/R injury results in a marked distortion and thinning of the neural retina.^10, 11, 35^ To assess the role of A2 in this I/R-induced tissue damage, we determined the impact of A2 deletion on retinal morphology at 7 days after I/R injury. Quantitative analysis of hematoxylin and eosin (H&E)-stained retinal sections using ImageJ (National Institute of Health, Bethesda, MD, USA) confirmed a significant thinning of the wild-type (WT) I/R retinas as compared with sham controls (Figure 2). However, A2 deletion preserved retinal morphology and prevented retinal thinning (Figures 2a and b). As the INL is highly sensitive to I/R injury, we also measured INL thickness in response to I/R injury. This analysis showed a 19% decrease in INL thickness in WT I/R retinas compared with sham controls, whereas INL thickness in A2^−/−^ I/R retinas was comparable to both sham control groups (Figure 2c). These data suggest that the I/R-induced increase in A2 expression is critically involved in mediating I/R-induced retinal injury.

### Reduction in retinal cell death and improved neuronal survival by A2 deletion

**Figure 3:**
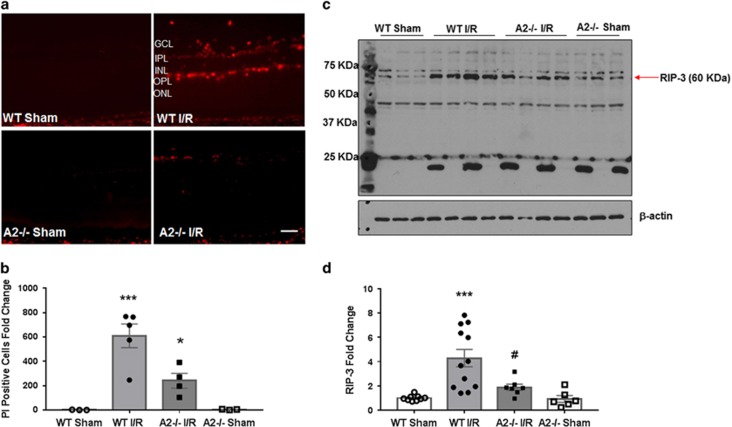
A2 deletion reduced IR injury-induced necroptotic cell death. (**a**) PI images and quantification (**b**) of PI-positive cells showing increases in necroptotic cells in the WT retina at 6 h after I/R injury. Results are presented as a fold change of WT sham. ***P<0.001 *versus* WT Sham, *P<0.05 *versus* WT I/R. WT sham and A2^−/−^ Sham: *n*=3, WT I/R: *n*=5, A2^−/−^ I/R: *n*=4. Scale bar 50 *μ*M. IPL, inner plexiform layer; OPL, outer plexiform layer; ONL, outer nuclear layer. Data are presented as mean±S.E.M. (**c**) Western blot and quantitative analysis (**d**) showing increases in RIP-3 expression in WT retina at 6 h after I/R injury. The red arrow points to the band of interest. Other bands are nonspecific. Molecular size marker is shown on the left. Results are presented as a fold change of WT sham. ***P<0.001 *versus* WT sham, ^#^*P*<0.05 *versus* WT I/R. WT sham: *n*=9, WT I/R: *n*=12, A2^−/−^ I/R: *n*=7, A2^−/−^ sham: *n*=6. Data are presented as mean±S.E.M.

The improvement in retinal morphology in the A2^−/−^ I/R mice suggested that A2 has a critical role in retinal injury during I/R. Previous studies in a rat model of I/R injury have shown that retinal neurons die by necroptosis, a caspase-independent mechanism of programmed cell death.^36, 37^ In this study, we examined the impact of A2 deletion on I/R-induced necroptosis by evaluating uptake of propidium iodide (PI) after I/R injury. Plasma membrane permeability is an early feature of necroptosis. PI is membrane impermeable in living cells, but when the plasma membrane is permeable it can enter the cell to bind to DNA. We observed a significant increase in PI-positive cells in WT retinas within 6 h following I/R injury. PI-labeled cells were mainly localized in GCL and INL of the I/R retinas (Figure 3a). The number of PI-positive cells in the inner retinal layers was significantly reduced with A2 deletion (Figure 3b). To further examine the role of necroptosis in I/R-induced injury, we assessed the expression of receptor interacting protein-3 (RIP-3) by western blotting. RIP-3 is a critical regulator of programmed necrosis/necroptosis.^38^ A significant increase in RIP-3 protein levels was evident at 6 h post-injury in WT I/R retinal lysates compared with sham controls (Figures 3c and d). The I/R-induced increase in RIP-3 was largely reduced in A2^−/−^ I/R retinas. These results suggest that A2 mediates I/R-induced cell death through a mechanism involving necroptosis.

**Figure 4:**
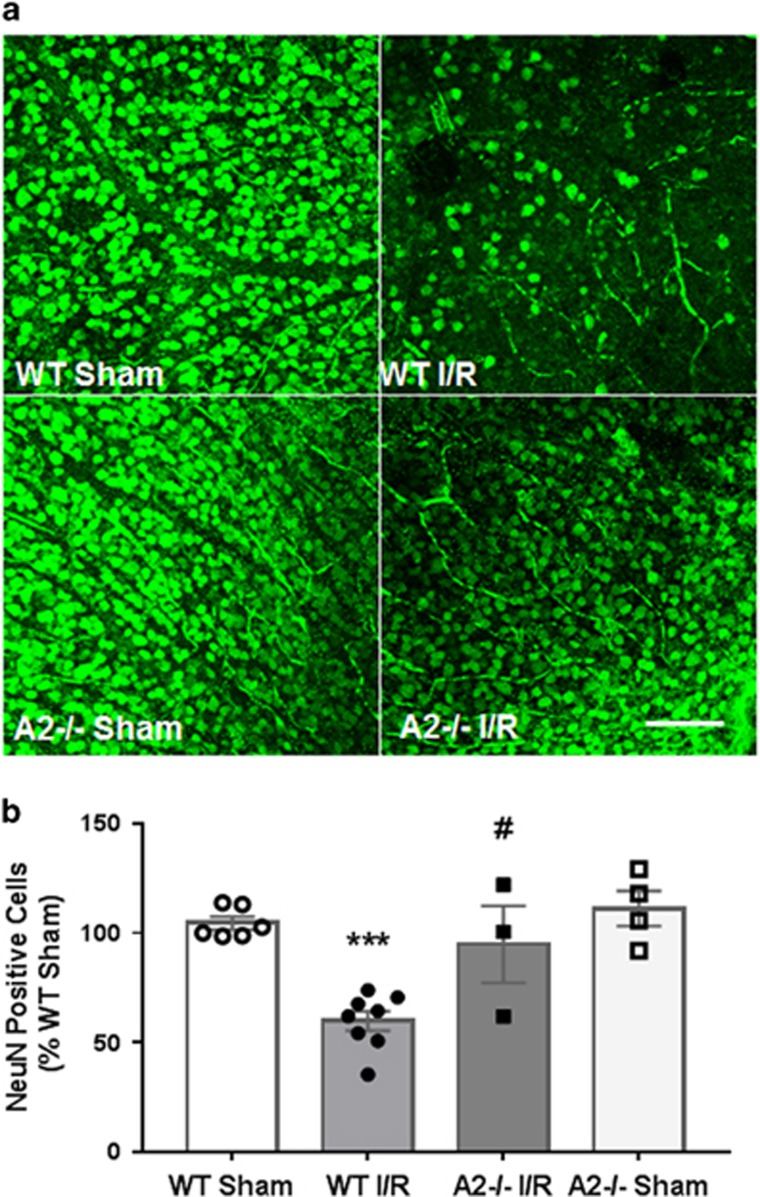
A2 deletion prevented I/R injury-induced loss of GCL neurons. (**a**) NeuN labeling and quantification (**b**) showing decreases in GCL neurons in WT retina at 7 days after I/R. Results are presented as a percentage of WT sham. ***P<0.001 *versus* WT sham, ^#^*P*<0.05 *versus* WT I/R. WT sham: *n*=6, WT I/R: *n*=8, A2^−/−^ I/R: *n*=3, A2^−/−^ sham: *n*=4. Scale bar 100 *μ*M. Data are presented as mean±S.E.M.

Degeneration of GCL neurons is another major hallmark of retinal damage following I/R insult.^7, 39, 40^ In order to assess the impact of A2 deletion on I/R-induced loss of GCL neurons, we prepared anti-NeuN immunolabeled retinal flat mounts from I/R treated WT and A2^−/−^ retinas and their respective sham controls. Consistent with our previous observations, there was a marked decrease in the number of NeuN-positive cells in the GCL layer of WT I/R retina compared to WT sham control at 7 days after I/R injury (Figure 4). Quantification using confocal imaging and ImageJ analysis showed a 40% decrease in GCL neurons in WT I/R retina compared with control. Deletion of A2 significantly blunted this effect (Figure 4b). The relative density of surviving GCL neurons in the A2^−/−^ I/R retina was not significantly different from that in the sham control retinas.

### Protection of the retinal microvasculature from I/R injury by A2 deletion

**Figure 5:**
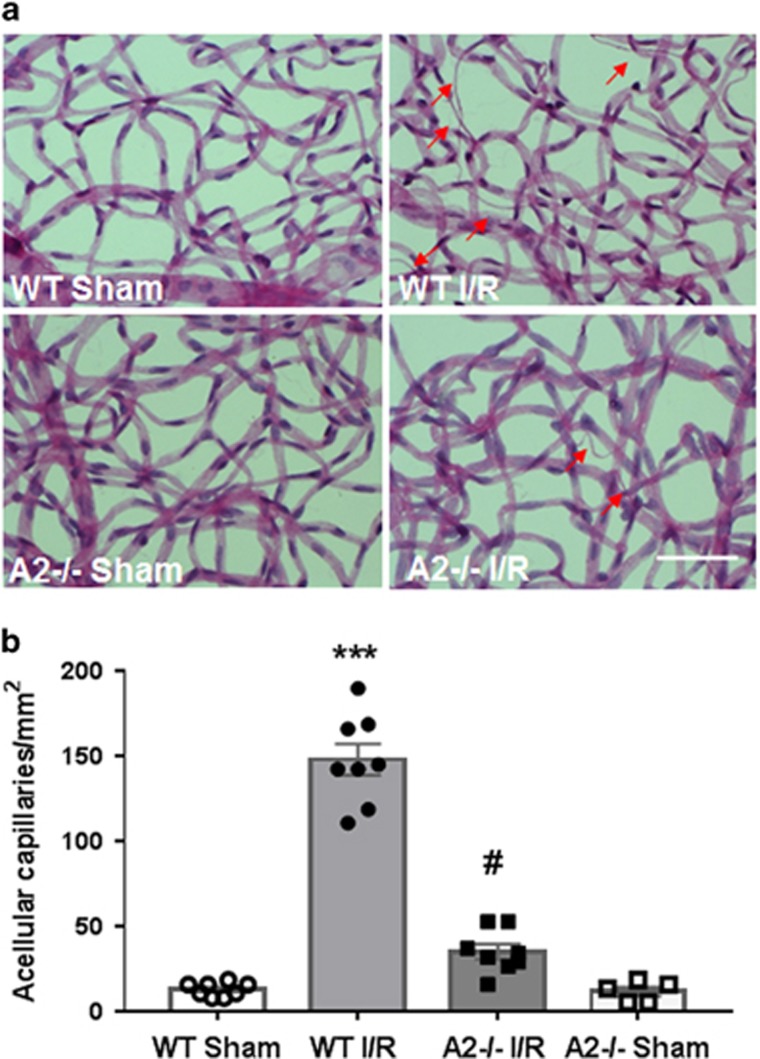
A2 deletion prevented I/R injury-induced microvascular degeneration. (**a**) Vascular digests and quantification (**b**) showing increases in acellular capillaries (arrows) in WT retina at 14 days after I/R injury. ***P<0.001 *versus* WT sham, ^#^*P*<0.001 *versus* WT I/R. A2^−/−^ sham: *n*=5, *n*=8 per group for others. Scale bar 50 *μ*M. Data are presented as mean±S.E.M.

In addition to the neuronal injury, I/R also induces degeneration of the retinal microvasculature. It has been reported that vascular cells start to die within 2 days after retinal I/R injury with maximum losses at 14 days.^7^ The vascular cell death leads to the formation of empty basement sleeves, which are termed acellular capillaries.^7^ Our previous study has shown that A2 deletion significantly reduced retinal vascular injury in the OIR mouse model.^15^ Based on these findings, we investigated the impact of A2 deletion on the formation of acellular capillaries after retinal I/R injury. We prepared vascular digests and quantified the number of acellular capillaries in bright field images using ImageJ software. This analysis showed a profound increase in the number of acellular capillaries (Figure 5a) in WT I/R retinas 14 days after I/R insult compared to their respective sham controls. The mean number of acellular capillaries in WT I/R vasculature is 148/mm^2^ and 13/mm^2^ in sham controls (Figure 5b). Deletion of A2 significantly reduced the formation of acellular capillaries in I/R retina (35/mm^2^).

### Reduction of glial activation and oxidative stress by A2 deletion

**Figure 6:**
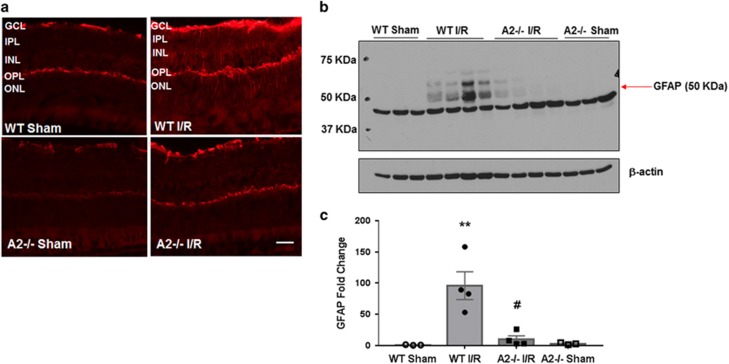
A2 deletion limited I/R injury-induced glial activation. (**a**) Immunofluorescence images showing increases in GFAP immunoreactivity in WT retina at 5 days after I/R injury. WT I/R: *n*=4, *n*=3 per group for others. Scale bar 50 *μ*M. IPL, inner plexiform layer; OPL, outer plexiform layer; ONL, outer nuclear layer. (**b**) Western blot analysis and quantification (**c**) showing increases in GFAP expression in WT retina at 5 days after I/R injury. The red arrow points to the band of interest. Other bands are nonspecific. Molecular size marker is shown on the left. Results are presented as a fold change of WT sham. **P<0.01 *versus* WT sham, ^#^*P*<0.01 *versus* WT I/R. WT Sham and A2^−/−^ sham: *n*=3, WT I/R and A2^−/−^ I/R: *n*=4. Data are presented as mean±S.E.M.

To further assess the effect of A2 deletion on I/R-induced retinal injury we examined glial cell activation by analyzing expression of glial fibrillary acidic protein (GFAP). GFAP is normally expressed in astrocytes and its upregulation is linked to retinal injury.^41^ Müller cells show increased GFAP expression under conditions of retinal injury and stress. We assessed GFAP expression by immunofluorescence and western blotting studies 5 days after injury. Immunolocalization analysis showed increased GFAP expression in astrocytes and Müller cells in WT I/R retinas compared with sham controls (Figure 6a). GFAP was markedly increased in Müller cells indicating their activation in response to I/R injury. GFAP expression in the A2^−/−^ retinas was largely unaffected by I/R injury. Western blot analysis further confirmed the immunolabeling results. GFAP levels were significantly increased in WT I/R retinas compared with sham controls. GFAP expression in the A2^−/−^ I/R retinas was markedly reduced as compared with WT I/R retinas and was similar to that in the sham controls (Figures 6b and c). These data suggest that A2 deletion protected against glial cell activation and limited the cell stress response following retinal I/R injury.

**Figure 7:**
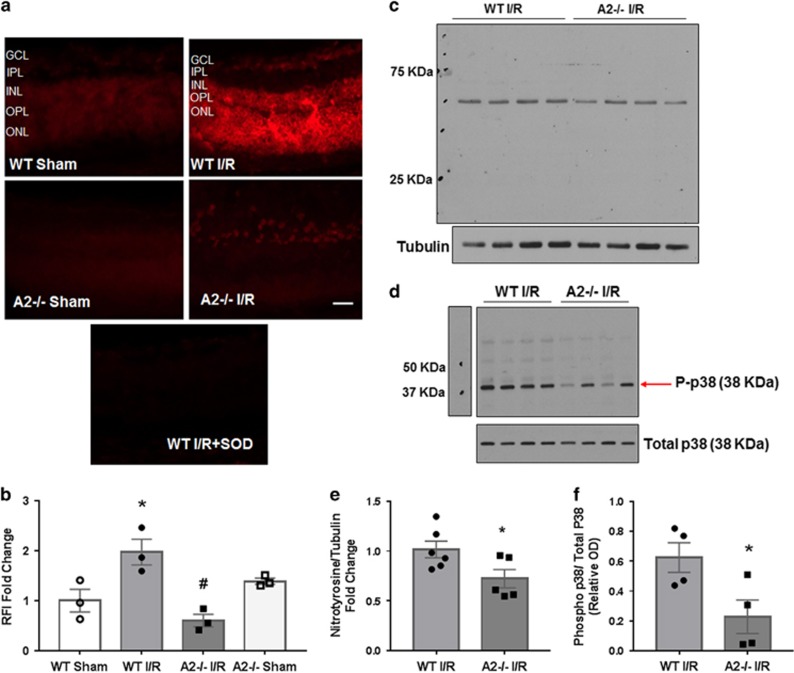
A2 deletion prevented I/R injury-induced oxidative stress. (**a**) DHE images of superoxide formation and quantification of the fluorescence intensity (**b**) showing increases in superoxide formation in WT retina at 6 h after I/R injury. Results are presented as a fold change of WT sham. *P<0.05 *versus* WT sham, ^#^*P*<0.01 *versus* WT I/R. *n*=3 per group for the four groups. Scale bar 50 *μ*M, RFI, relative fluorescence intensity. IPL, inner plexiform layer; OPL, outer plexiform layer; ONL, outer nuclear layer. Data are presented as mean±S.E.M. (**c**) Western blot analysis with quantification (**e**) showing decreases in nitrotyrosine formation in A2^−/−^ retina at 6 h after I/R injury. Molecular size marker is shown on the left. Results are presented as fold change of WT I/R. *P<0.05 *versus* WT I/R. WT I/R: *n*=6, A2^−/−^ I/R: *n*=5. Data are presented as mean±S.E.M. (**d**) Western blot analysis with quantification (**f**) showing decreses in p38 phosphorylation in A2^−/−^ retina at 3 h after I/R injury. The red arrow points to the band of interest. Other bands are non specific. Molecular size marker is shown on the left. *P<0.05 vs WT I/R. *n*=4 per group for the two groups; OD, optical density. Data are presented as mean±S.E.M.

Oxidative stress is another key mediator of I/R-induced retinal neurovascular injury. In order to assess the involvement of A2 in I/R-induced oxidative stress, we determined the effects of A2 deletion on the formation of superoxide and peroxynitrite (ONOO^−^). We used dihydroethidium (DHE) imaging of fresh frozen retinal sections to assess superoxide formation. When superoxide is produced it binds to DHE and oxidizes it to ethidium, which binds to DNA and fluoresces red. We have previously shown that 6 h after I/R injury there is a significant increase in superoxide formation in WT retinas.^11^ This was confirmed in our present study. Quantification of DHE assay images using fluorescence microscopy and Metamorph imaging system analysis showed that the I/R injury induced a significant increase in superoxide formation in the WT retinas and that this increase was largely prevented in the A2^−/−^ retinas (Figures 7a and b). As DHE can be oxidized by other reactive oxygen species (ROS) in addition to superoxide,^42^ we performed control studies using superoxide dismutase (SOD) to demonstrate specificity of the DHE reaction for superoxide.

Another marker of oxidative stress is ONOO^−^. ONOO^−^ is a short-lived molecule at physiological pH, but it has been shown to nitrate protein tyrosine residues and thus can serve as a ONOO^−^ biomarker. We have previously shown that nitrotyrosine formation is significantly increased 6 h after I/R injury.^11^ Therefore, we examined ONOO^−^ formation indirectly by western blotting analysis with an anti-nitrotyrosine antibody. A significant reduction in the level of nitrated proteins was observed in A2^−/−^ I/R retina compared with WT I/R (Figures 7c and d). These findings further support the involvement of A2 in I/R injury-induced oxidative stress.

The p38 mitogen-activated protein kinase is one of the mitogen-activated protein kinases that is activated in response to cell stress. Phosphorylation of p38 is an indicator of its activation. We used western blotting to assess p38 phosphorylation in samples collected 3 h after injury and observed a significant increase in phospho-p38 in WT I/R retinal lysates compared with sham controls (Figures 7e and f). Phosphorylation of p38 was significantly reduced in A2^−/−^ I/R retinas compared with WT I/R retinas.

### Protection against I/R-induced impairment of retinal function by A2 deletion

**Figure 8:**
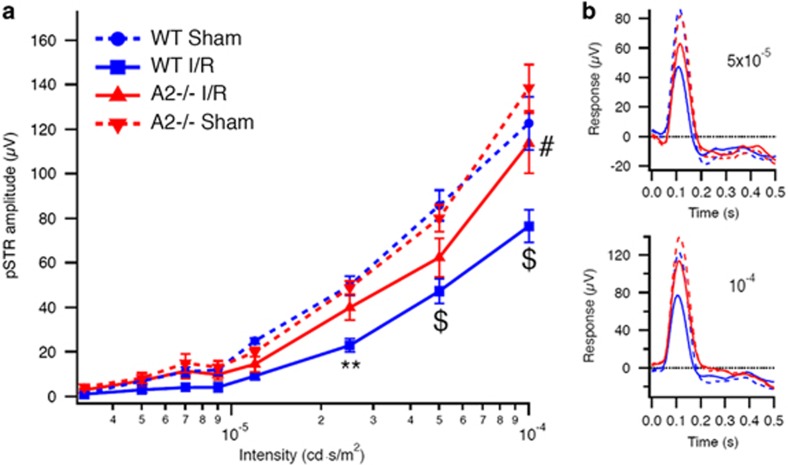
A2 deletion prevented I/R injury-induced impairment of inner retina function. (**a**) Amplitudes of the pSTRs are plotted against stimulus intensity showing impaired function of the inner retinal neurons at 4 weeks after I/R injury. **P<0.01 *versus* WT sham, ^$^*P*<0.0001 *versus* WT sham, ^#^*P*<0.0001 *versus* WT I/R. WT sham and WT I/R: *n*=7, A2^−/−^ sham and A2^−/−^ I/R: *n*=8. Data are presented as mean±S.E.M. (**b**) Averaged responses to 5 ms flashes are shown for the two highest intensities in panel **a** showing improved response of A2^−/−^ I/R at 5 × 10^−5^ cd.s/m^2^ (top panel) and significant protection at 10^−4^ cd.s/m^2^ (bottom panel).The pSTR amplitude was measured at 110 ms after the flash

To assess the effect of A2 deletion on function of the inner retina, we used dark-adapted (scotopic) electroretinography (ERG) to record the positive scotopic threshold response (pSTR). The pSTR is thought to reflect activity of the proximal retinal portion, that is, amacrine and ganglion cells, of the sensitive rod circuit.^43^ This analysis showed that the amplitude of the pSTR was significantly reduced in WT I/R mice compared with WT sham 4 weeks after the injury (Figure 8). This I/R-induced impairment of the pSTR was significantly lessened in the A2^−/−^ I/R retinas at higher intensity stimulation.

## Discussion

In this study, we demonstrate for the first time that A2 enzyme is crucially involved in I/R-induced neurovascular degeneration. We show that deletion of A2 gene preserved retinal morphology, improved neuronal survival and decreased the formation of acellular capillaries while reducing oxidative stress, gliosis, phosphorylation of p38 MAPK and necroptosis-mediated cell death following I/R injury. These protective effects were associated with an improvement in function of the inner retinal neurons as shown by ERG analysis.

**Figure 9:**
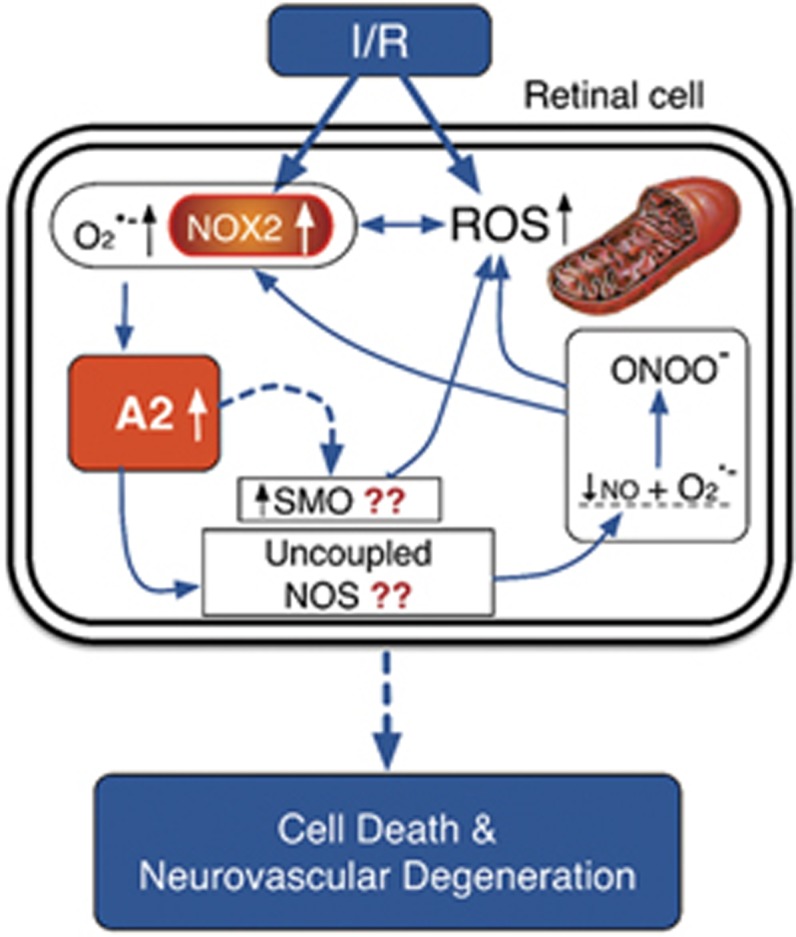
Possible mechanisms of neurovascular degeneration after retinal I/R. Cartoon showing possible sources of superoxide and other ROS, which can increase A2 expression. A2 upregulation depletes l-arginine needed for NO production by NOS. This leads to NOS uncoupling with production of more superoxide, which reacts with NO to form ONOO^−^, further increasing ROS levels. A2 upregulation can also increase SMO expression and activity, further increasing ROS production. These mechanisms can activate cell death pathways leading to neurovascular degeneration after retinal I/R insult. O_2_^.−^, superoxide; NOX2, NADPH oxidase 2; SMO, spermine oxidase; ONOO-, peroxynitrite; NOS, nitric oxide synthase; NO, nitric oxide. Question marks point to potential mechanisms that will be investigated in future studies

Our previous studies in the mouse model of I/R injury have shown that expression and activity of NOX2 NADPH oxidase are involved in the neuronal cell death. We found that NOX2 deletion reduced I/R-induced increases in oxidative stress, gliosis and activation of cell stress pathways and protected against neuronal loss.^11^ Interestingly, western blot analyses of retinal protein samples from the same animals showed that levels of the A2 protein were increased in the WT retina after I/R injury. This increase was completely prevented by deletion of NOX2, suggesting that A2 is a downstream target of NOX2-induced increases in oxidative stress (unpublished data, H Yokota and RB Caldwell). The mechanism by which NOX2 activation increases expression of A2 is unclear. However, we believe that superoxide produced from activated NOX2 along with other ROS, which are markedly increased after I/R has an important role in A2 upregulation that leads eventually to cell death and I/R-induced neurovascular degeneration (Figure 9). Relatively little is known about regulation of A2 expression. However, previous studies have shown that oxidative species can increase A1 expression in vascular endothelial cells through protein kinase C-mediated activation of the RhoA/Rho kinase pathway.^44^ Studies using mesenteric arteries from streptozotocin-induced diabetic rats have shown a link between increased formation of phospho-p38 MAPK, increased expression of A1 and A2 and vascular dysfunction.^45^ These effects were reversed by p38 MAPK inhibition. A mechanism involving inflammation is also possible given that A2 expression in macrophages has been shown to be increased by liver X receptor.^46^

Arginase has been identified as a potential therapeutic target for treatment of coronary artery disease (CAD) in a rat model.^25^ An arginase inhibitor has been shown to protect against endothelial dysfunction caused by ischemia reperfusion in patients with CAD.^30^ Recent reports have implicated A2 in endothelial dysfunction through endothelial nitric oxide synthase (eNOS) uncoupling in obese and aged mice,^32, 33^ as well as in impairment of cerebral blood flow after TBI.^34^ In addition, recent studies from our laboratory have demonstrated the involvement of A2 in neurovascular damage in a neonatal mouse model of ischemic retinopathy.^13, 14, 15^

In this study, we demonstrate a significant increase in A2 mRNA and protein levels as early as 3 h after I/R insult. This early elevation of A2 expression implies a primary role in the pathological process. Although the role of arginase in retinal I/R injury has not been studied until now, our previous studies have shown that both arginase isoforms are expressed in the retina.^12^ We also have found that streptozotocin-induced diabetes causes an increase in retinal arginase activity^12, 47^ and that inhibition of arginase activity or deletion of one copy of A1 and both copies of A2 reduces diabetes-induced retinal inflammation.^12^ Furthermore, our studies in a mouse model of OIR have shown that homozygous deletion of A2 prevents hyperoxia-induced neuronal-glial injury and improves retinal function.^13^ Studies in the same model showed that A2 deletion also prevents oxidative stress and limits retinal vascular degeneration and pathological neovascularization.^15^ We have shown that A2 is highly expressed in the horizontal cells and in the ONL in OIR model.^13, 14^ A2-induced cell death in other retinal layers could be mediated through signals initiated by cell–cell interactions or release of soluble cytotoxic factors from activated cell types such as micro- or macro-glia. This will be investigated in future studies.

The role of oxidative stress and inflammation in neuronal cell loss and formation of acellular capillaries after I/R insult in retina is well established.^11, 48, 49^ Furthermore, studies have shown that the transcription factor NF-E2-related factor 2 has a significant role in protecting cells from I/R-induced neurodegeneration by a mechanism involving decreases in oxidative stress.^50^ We have previously shown that A2 deletion prevented oxidative stress and reduced hyperoxia-induced retinal vascular degeneration in a model of OIR.^15^ Our group has also shown that retinal I/R insult significantly increases superoxide and nitrotyrosine formation by mechanisms involving activation of NOX2.^11^ In this study, we are introducing A2 as a new factor in mediating oxidative stress and neurovascular degeneration following retinal I/R injury. We show that deletion of A2 significantly reduced the I/R-induced increases in superoxide and nitrotyrosine formation. Taken together with our finding that deletion of NOX2 limits I/R injury induced increases in A2 expression, the latter result suggests that NOX2-induced increases in oxidative stress are due in part to the upregulation of A2. It should be noted that there is a difference in relative intensity of the DHE reaction in the INL and GCL, which is consistent with the patterns of PI labeling in these layers. In contrast, the lack of PI labeling in the ONL in combination with the intense DHE reaction suggests that the photoreceptors are dying at a different time and/or are less sensitive to oxidative injury than the INL and GCL neurons.

Further studies are needed to elucidate the mechanisms by which A2 increases oxidative stress. However, our investigations in models of OIR and diabetic retinopathy suggest that increases in superoxide and ONOO^−^ formation subsequent to arginase-induced depletion of the l-arginine supply to nitric oxide synthase (NOS) may be involved (Figure 9).^12, 15, 47^ Our studies in the OIR model have also identified a downstream element in the arginase pathway, altered polyamine metabolism by spermine oxidase (SMO), as a source of oxidative stress and damage to retinal neurons.^14^ Oxidation of spermine by SMO has been shown to lead to production of hydrogen peroxide and the reactive aldehyde 3-amino propanal.^51^ Both can damage DNA, RNA, proteins and membranes. Our studies in the OIR model have shown that hyperoxia-induced neuronal cell death is associated with increase in SMO expression along with elevated levels of hydrogen peroxide formation and that treatment with a SMO inhibitor reduced oxidative stress and decreased neuronal injury.^14^ It is possible that the neurovascular protection from I/R injury provided by A2 deletion is mediated via disruption of the polyamine oxidase signaling pathway. This will be investigated in our future studies (Figure 9).

Activation of Müller glial cells, characterized by increased GFAP expression, is a well-established injury marker in retinal disease conditions such as I/R, diabetic retinopathy and OIR.^13, 52, 53^ This was confirmed in our study. Further, the I/R-induced activation of Muller cells was prevented by A2 deletion, demonstrating a protective effect of A2 deletion on glia. This is consistent with our previous observation that A2 deletion abrogates glial activation in the OIR model.^13^

Death of neurons in the inner retina following I/R can occur through different mechanisms including apoptosis, necrosis and programmed necrosis, that is, necroptosis^36, 37^ In this study, PI labeling showed a significant increase in PI-positive cells in the inner retina at 6 h after I/R. The number of PI-positive cells was significantly reduced in the retinas of A2 deficient mice. PI staining showed a reduced signal in the GCL compared with the INL in WT retinas. However, NeuN labeling showed a severe loss of GCL neurons. This could mean that GCL neurons are dying by other mechanism and/or at different time points. PI labeling was done 6 h after I/R, but neurodegeneration was assessed 7 days after I/R. It should be noted that while PI labels cells dying by necroptosis, it also stains late apoptotic and early necrotic cells. Thus, we examined expression of RIP-3, a marker for necroptotic cell death. Previous studies have reported that RIP-3 expression is rapidly increased in cells of the GCL and INL in the rat retina following acute increases in intraocular pressure (IOP).^54^ It was also reported that RIP-3 immunolabeling was colocalized with the PI-positive cells in the GCL that indicates the involvement of RIP-3 in necroptotic cell death. Our western blotting analysis showed a significant increase in RIP-3 protein expression following I/R, which was significantly reduced in A2-deficient retinas. These results indicate that A2 is involved in the I/R-induced necroptosis.

In conclusion, our study demonstrates for the first time that A2 is crucially involved in I/R-induced retinal neurovascular injury, through a mechanism involving increased oxidative stress, glial activation and necroptotic cell death. Our study suggests that A2 can be considered as a therapeutic target to decrease neurovascular degeneration after I/R injury.

## Materials and Methods

### Animals and I/R insult

All procedures with animals were performed in accordance with the ARVO Statement for the Use of Animals in Ophthalmic and Vision Research and were approved by the institutional animal care and use committee (Animal Welfare Assurance no. A3307–01). All surgeries were performed under anesthesia, and all efforts were made to minimize suffering. We used male WT and A2-deficient (A2^−/−^) mice on C57BL6J background. These mice were subjected to I/R injury in the right eye. I/R was induced as previously described.^11^ Briefly, mice (10–12 weeks old) were anesthetized with (73 mg/kg ketamine hydrochloride and 7.3 mg/kg xylaxine hydrochloride, i.p.), 1% tropicamide (Akorn, Lake Forest, IL, USA) was used to dilate the pupil, and topical anesthesia (0.5% proparacaine hydrochloride; Akorn) was applied to the cornea. A 30-gauge needle cannulated in the anterior chamber of the right eye was used to infuse sterile saline. The IOP was raised to 110 mm Hg by elevating the saline reservoir and this pressure was held for 40 min. Ischemia was confirmed by observation of whitening of the anterior segment of the globe and blanching of episcleral veins.^55^ The left eye was used as control. The mice were killed at various times after I/R as determined in preliminary studies and the existing literature as follows:


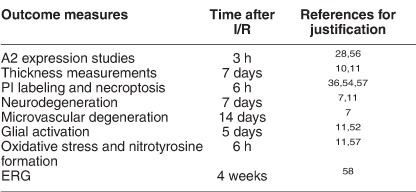


### Quantitative RT-PCR

Total RNA was extracted from frozen retinal tissue using a RNA isolation kit (Invitrogen, Carlsbad, CA, USA) as instructed by the manufacturer. RNA was converted to cDNA using M-MLV reverse transcriptase (Invitrogen). Quantitative PCR was performed using an ABI StepOne Plus Thermocycler (Applied Biosystems, Foster City, CA, USA) with TaqMan gene expression assays (Invitrogen). The probes of TaqMan assay used to detect A1, A2 and hypoxanthine phosphoribosyltransferase (HPRT) as internal control, were Mm00475988_m1, Mm00477592_m1 and Mm00446968_m1. Data were normalized to HPRT and the fold change between levels of different transcripts was calculated by the CT method.

### Neurodegeneration evaluation

Neuronal degeneration was assessed as previously described with minor modification.^11^ The neuronal cell marker NeuN was used to label surviving neurons in whole retinal flat mounts. Eye balls were collected 7 days after I/R or sham surgery and fixed overnight in 4% paraformaldehyde (PFA) at 4 °C. Retinas were dissected, permeabilized, blocked and then labeled with anti-NeuN (Millipore cat. # MAB377, Billerica, MA, USA) in 37 °C for 2 h. Then retinas were incubated with Alexa488 anti-mouse IgG overnight. After flatmounting, retinas were imaged using a confocal microscope (LSM 510; Carl Zeiss, Thornwood, NY, USA). Four images were taken in the midperiphery of each retina and the NeuN-positive cells were counted using ImageJ software. The result was presented as a percentage of NeuN-positive cell numbers in the ganglion cell layer (GCL) of the I/R eyes compared with the sham eyes.

### Retinal vasculature isolation and measurement of acellular capillaries

Trypsin digestion method was used for isolating the retinal vasculature as previously described with minor modification.^59^ Eye balls were removed and fixed with 4% paraformaldehyde overnight. Retinas were carefully dissected and incubated in distilled water at room temperature with gentle shaking at least 24 h. Then the retinas were digested with 3% trypsin (Difco Trypsin 250, Becton Dickinson and Company, Sparks, MD, USA) in 0.1 M Tris buffer (pH 7.8) for 1.5 h at 37 °C on an orbital shaker (50 r.p.m.). Retinas were washed carefully in several changes of fresh distilled water until no more non-vascular tissue was observed under the microscope. The isolated retinal vasculature was air-dried on silane-coated slides and stained with periodic acid–Schiff and hemotoxylin. Acellular capillaries were counted in 10 random areas of the mid-retina. The field area was calculated using ImageJ software. The number of acellular capillaries were divided by the field area to get number of acellular capillaries per 1 mm^2^ of retina.

### Retinal thickness measurement

Retinal thickness was studied on retinal frozen sections from WT and A2−/− animals 7 days after the I/R injury. Cross-sections with optic nerve attachment were prepared (10 *μ*m) followed by H&E staining for morphological observation. Images were taken 162 *μ*m away from the optic nerve head and four sections 20 *μ*m apart from each other were used. Whole retina or individual retinal layer (inner nuclear layer, INL) thicknesses at three different distances from optic nerve head was determined using ImageJ software and averaged. Averaged retinal thickness was presented as percentage compared with the contralateral sham eyes.

### ROS production

Dihydroethedium (DHE) method was used to evaluate superoxide formation as described previously.^15^ Briefly, fresh frozen sections were preincubated with or without SOD-polyethylene glycol (400 U/ml, Sigma-Aldrich, St. Louis, MO, USA) for 30 min, followed by reaction with DHE (10 *μ*M) for 15 min at 37 °C. Superoxide oxidizes DHE to ethidium bromide, which binds to DNA and fluoresces red.^60^ The fluorescence microscope (AxioVision; Carl Zeiss) was used to obtain the DHE images immediately after incubation. DHE was excited at 488 nm with an emission spectrum of 610 nm. Six images per slide were taken and computer-assisted morphometry (Metamorph Image System; Molecular Devices, Sunnyvale, CA, USA) was used to analyze images for fluorescence intensity.

### Immunofluorescence

Eyes were enucleated, fixed in 4% PFA (overnight, 4 °C). The next day, eye balls were washed in PBS and then incubated with 30% sucrose overnight at 4 °C. Then eye balls were snap frozen in optimal cutting temperature (OCT) solution. Cryostat sections (10 *μ*m) were obtained, mounted on glass slides, permeabilized with 1% Triton for 10 min and blocked in 10% normal goat serum for 1 h. Sections were then incubated in anti-GFAP (Dako Cat. # Z0334, Carpinteria, CA, USA; 1 : 200) primary antibody at 4 °C overnight. On the next day, sections were incubated for 1 h at room temperature in fluorescein-conjugated secondary antibody (Molecular Probes, Grand Island, NY, USA; 1 : 500), washed in PBS, and covered with mounting medium and DAPI (Vectashield; Vector Laboratories, Burlingame, CA, USA).

### Western blot analysis

Retinal protein extracts were prepared using RIPA buffer (Millipore) containing 1x protease and phosphatase inhibitors (Complete Mini and phosSTOP, respectively; Roche Applied Science, Indianapolis, IN, USA). We separated the proteins on SDS-PAGE and then transferred them to nitrocellulose membrane (Millipore) blocked in 5% milk (Bio-Rad, Hercules, CA, USA). The membranes were then incubated with different primary antibodies overnight at 4 °C. The primary antibodies we used are: A2 (Santa Cruz Biotechnology cat. # Sc-20151, Dallas, TX, USA; 1 : 500), phospho-p38 (Cell Signaling Technology cat. # 4511, Danvers, MA, USA; 1 : 500), total p38 (Cell Signaling Technology cat. # 9212, 1 : 500) GFAP (Sigma-Aldrich cat. # G6171, St. Louis, MO, USA; 1 : 500), RIP-3 (Santa Cruz Biotechnology cat. # SC-135170, 1 : 500), tubulin (Sigma-Aldrich cat. # T-9026, 1 : 5000) and *β*-actin (Sigma-Aldrich cat. # A1978, 1 : 5000). Primary antibodies were diluted in either 5% milk or 5% bovine serum albumin (BSA). ONOO^−^ formation was determined indirectly by western blot analysis of nitrotyrosine using monoclonal anti-nitrotyrosine antibody (Millipore Cat. # 05-233, 1 : 500 in 1% BSA). The next day, membranes were washed in TBST (Tris-buffered saline with 0.5% Tween-20) and horseradish peroxidase-conjugated secondary antibodies (GE Healthcare, Piscataway, NJ, USA) were added (1 : 5000 for tubulin and actin, 1 : 4000 for nitrotyrosine and 1 : 1000 for others). Enhanced chemiluminescence system (GE Healthcare Bio-Science Corp., Piscataway, NJ, USA) was used to detect immunoreactive proteins. Data were quantified by densitometry using ImageJ and normalized to loading control.

### PI labeling and detection of necrotic cells

PI was used to label necrotic cells as previously described.^36^ Briefly, mice were subjected to I/R injury. At 5 h after the I/R injury, PI (Sigma-Aldrich Cat. #p4170, St. Louis, MO, USA, 5 mg/kg) was injected intraperitoneally and mice were killed 1 h later by carbon dioxide inhalation followed by cervical dislocation. Eye balls were harvested and snap frozen in OCT compound. For detecting PI-positive cells, retinal sections (10 *μ*m) were fixed in 100% ethanol for 10 min at room temperature and quantification of necrotic cells was performed on three sections from each sample using a Carl Zeiss Anxioplan2 fluorescence microscope.

### Electroretinogram

Mice were dark-adapted overnight, anesthetized with ketamine/xylazine and prepared under dim red lighting. The eyes were treated with drops of proparacaine, tropicamide and phenylephrine. A rectal probe controlled a heating pad to maintain temperature at 37 °C. A ground electrode was placed in the tail, and reference electrodes in each cheek. Silver thread electrodes were placed on each eye, and a drop of hypromellose was added to improve electrical contact and protect the cornea from drying. An optic fiber was then positioned just in front of each pupil. Visual stimuli were generated by an LED device, with the light from the LED defocused and filtered before arriving at the optic fiber launcher in order to provide extremely dim luminances, ranging from about 10^−6^ to 10^−4^ candela.second/meter^2^ (cd.s/m^2^). Testing consisted of a set of 5 ms flashes over a range of intensities, randomly interleaved with a probability distribution emphasizing intensities just above threshold (which is around 4 × 10^−6^ cd.s/m^2^). Responses were averaged over 10–100 trials at each intensity, and positive (pSTR) and negative (nSTR) scotopic threshold responses were measured at 110 and 200 ms, respectively^43^ after the flash that occurred 500 ms into each 2-s trial. The pSTR and nSTR amplitudes had floors at 0 *μ*V. The results were averaged across the mice in each group (WT and A2 −/−), and the differences between the sham and I/R eyes were used to estimate the effects of the knockout on the damage caused by the I/R.

### Statistical analysis

Results were presented as mean±S.E.M. Statistical analysis was performed by GraphPad Prism 7 (GraphPad Softwar Inc., La Jolla, CA, USA). Values were tested to assess whether they followed a normal distribution by the same software. Two-way ANOVA followed by Tukey's test for multiple comparisons or, the Student's *t*-test (two-tailed) in case of single comparisons, were used. *P*≤0.05 was considered statistically significant. For ERG studies, two-way ANOVAs were computed to gauge the effects of the genotype across stimulus intensities, and the effects at individual intensities were assessed by *t*-tests after Holm–Bonferroni correction for the multiple comparisons.

## Abbreviations

I/R: ischemia/reperfusion
A1: arginase 1
A2: arginase 2
AD: Alzheimer's disease
TBI: traumatic brain injury
WT: wild-type
A2^−/−^: arginase 2-deficient mice
IOP: intraocular pressure
HPRT: hypoxanthine phosphoribosyltransferase
PFA: paraformaldehyde
GCL: ganglion cell layer
H&E: hematoxylin and eosin
INL: inner nuclear layer
DHE: dihydroethedium
OCT: optimal cutting temperature
BSA: bovine serum albumin
PI: propidium iodide
ERG: electroretinogram
pSTR: positive scotopic threshold response
nSTR: negative scotopic threshold response
OIR: oxygen-induced retinopathy
RIP-3: receptor interacting protein kinase-3
GFAP: glial fibrillary acidic protein
ROS: reactive oxygen species
SOD: superoxide dismutase
ONOO^−^: peroxynitrite
CAD: coronary artery disease
SMO: spermine oxidase
eNOS: endothelial nitric oxide synthase
NOS: nitric oxide synthase
